# Angioedema: Is ICU admission warranted? A single institution assessment

**DOI:** 10.2478/jccm-2025-0023

**Published:** 2025-07-31

**Authors:** Madeleine Brill-Edwards, W. Chase Hamilton, Erika J. Yoo, Jennifer Costello, George J. Koenig, Murray J. Cohen, Joshua A. Marks

**Affiliations:** Eastern Virginia Medical School, Norfolk, VA, USA; Department of Anesthesia, Sidney Kimmel Medical College at Thomas Jefferson University, Philadelphia, PA USA; Bethlehem Campus, St. Luke's University Health Network, Fountain Hill, PA, USA; Division of Pulmonary, Allergy and Critical Care Medicine, Jefferson-National Jewish Health Jane and Leonard Korman Respiratory Institute, Sidney Kimmel Medical College at Thomas Jefferson University, Philadelphia, PA, USA; Division of Acute Care Surgery, Department of Surgery, Sidney Kimmel Medical College at Thomas Jefferson University, Philadelphia, PA USA

**Keywords:** angioedema, airway management, ICU resources, hospital resource allocation, critical care

## Abstract

**Introduction:**

Angioedema has potential for rapid airway decompensation requiring intervention. Patients are often admitted to an ICU for “airway watch.” There is a lack of evidence to support which patients require this.

**Aim:**

We aimed to characterize admission patterns and outcomes of angioedema patients at our institution to assess resource utilization and necessity of ICU use. We hypothesized that patients not requiring intubation upon presentation are safe to manage outside the ICU.

**Materials and Methods:**

Retrospective chart review of patients admitted to our urban academic quaternary referral institution with angioedema ICD-10 codes between 2017 and 2020. Charts reviewed for demographics, etiology, admission location, level of care, length of stay (LOS), intubation information, discharge destination, and specific treatment administered. Statistical analysis included a t-test for continuous variables (LOS).

**Results:**

Of 135 encounters for angioedema, 117 patients were admitted. 50 were admitted to an ICU. Patients were evenly split based on sex, majority black, and the most common etiology was ACE-inhibitor use. 20 required airway intervention with intubations primarily outside the ICU setting and only 2 in the ICU. 1 surgical airway performed in the ED. The mean time from presentation to intubation was 2.7 hours (Min 0h; Max 7.5h). The average ICU LOS for non-intubated patients was 1.1 days, with hospital LOS 1.5 days compared to 0.25 days for those not admitted to an ICU (p<0.001). For intubated patients, average ICU LOS was 4.3 days, with hospital LOS 6.2 days. All intubated patients were successfully liberated from the ventilator. No deaths occurred.

**Conclusion:**

Most angioedema encounters did not require airway intervention within the first hours of presentation. Airway decompensation and intervention mostly occurred prior to the ICU setting. ICU resources should be carefully allocated and may be unnecessary for patients presenting with angioedema who are not intubated on initial evaluation.

## Introduction

Angioedema is a common presentation in the ED with more than one million visits a year. Although studies like Sandefur et al. report that 70% of angioedema patients are discharged from the ED, there is still the very serious risk of rapid airway decompensation. Angioedema is a disease process with various etiologies that results in nonpitting, nondependent, asymmetric edema of the subcutaneous and submucosal tissues. Different etiologies include histamine-mediated which is allergic in nature, bradykinin-mediated which can be more severe and longer lasting, and less commonly hereditary or acquired angioedema that is mediated by complement pathway abnormality. The intubation rate for airway compromise in angioedema patients is estimated to be between 3 to 11% according to literature review. While this is a relatively uncommon complication, this risk can be the reason for admission of angioedema patients to the Intensive Care Unit (ICU) for observation, which is informally called “airway watch” [1,2,3,4,5].

There is limited evidence to support which patients benefit from “airway watch”. One factor that may play a role in these decisions includes determination of the etiology of angioedema, which can aid in management and treatment. Angiotensin-converting enzyme inhibitors (ACEi) are the most common causative agent via a bradykinin mediate pathway (approximately one third of patients presenting to Emergency Department [ED]), followed by nonsteroidal anti-inflammatories (NSAIDs). Conversely, there are a large number (30–59%) of angioedema cases of unknown etiology which poses a challenge in triage and management. There are also several triage tools and staging criteria in development to aid in decision making. This includes nasopharyngeal laryngoscopy (NPL) evaluation by Otolaryngology (ENT) or emergency medicine providers for predictive anatomical findings such as laryngeal or lingual edema as outlined by Ischoo et al. However, there is no one tool or criteria that has been validated or universally accepted for triaging these patients. As for epidemiology as a decision tool, it has been observed that there is a higher incidence of ACEi angioedema in African Americans, women, and smokers as well as a higher susceptibility for requiring emergent airways in African American women, but the underlying mechanism for these observations is not well understood.

Because of this uncertainty and lack of data to support clinical decision making, we sought to characterize admission patterns and outcomes of patients with angioedema at our urban quaternary referral center to assess the necessity of ICU level of care in “airway watch” patients and utilization of ICU resources. We hypothesized that patients with angioedema not requiring intubation upon presentation would be safe to manage outside of the ICU [2,3,5,6,7,8].

## Materials and Methods

**Fig. 1.**
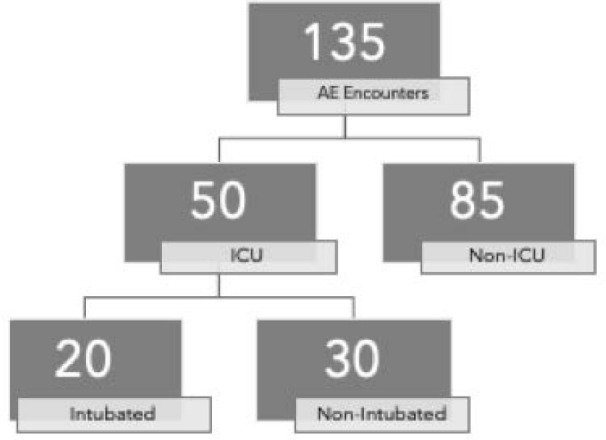
Breakdown of Angioedema (AE) admission results with locations including the Intensive Care Unit (ICU) and non-ICU, as well as intubated and non-intubated status upon ICU admission.

We completed a retrospective chart review of patients admitted for angioedema based on ICD-10 codes (T78.3, T78.3XXA, D72.11, D84.1) at Thomas Jefferson University Hospital (TJUH), an urban quaternary referral institution, between 2017 and 2020. Patients were grouped into non-ICU level of care and ICU level of care (Table 1). The ICU encounters were further sorted into intubated and non-intubated groups (Table 2, Figure 1). Data collection included: demographics (recorded race, sex, age), etiology of angioedema (if known), admission location, length of stay (LOS) in the ICU and non-ICU setting, preexisting comorbidities (obstructive sleep apnea, lung disease), diagnosis of hereditary angioedema or prior episodes, intubation information (location, timing, rationale), ventilator days, ENT consultation, specific treatment administered, and discharge destination. The primary outcome measured was the need for intubation. We further gathered details regarding intubation setting and timing. Secondary outcomes included hospital and ICU LOS. Descriptive statistics were performed using t-test for continuous variables including comparison of LOS. Categorical variables were compared using odds ratios with univariate analysis.

**Table 1.** Overall Patient Characteristics & Demographic, Non-ICU vs ICU

	**Overall**	**Non-ICU**	**ICU**
Encounters	135	85	50
Patients	117	69	48

**Demographics**			
Mean Age (years)	58.5	56.0	61.9
Sex	M, 63, 55%	M, 39	M, 24
	F, 54, 45%	F, 30	F, 24
Race			
Black/AA	89, 66%	57	32
White	39, 29%	25	14
Other	7, 5%	3	4

**Etiology of AE**			
	Unknown 63, 47%	Unknown, 48	ACEi, 27
	ACEi 51, 38%	ACEi, 26	Unknown, 15
	Other, 13, 10%	Other, 7	Other, 6
	Hereditary 6, 5%	Hereditary, 4	Hereditary, 2

**Mean LOS (days)**			
Total Hospital LOS	1.43 2.9	0.31 0.69	3.37
ICU LOS	--	--	2.40

**Treatment**			
Histamine/corticosteroid/epinephrine regimen	120, 78.5%	72	48
Biologic Agent (Icanibant)	1, 1%	1	0
Other/No treatment	14, 9.5%	12	2

**Otolaryngology (ENT)**			
Had ENT Consult	99, 73.3%	50, 58%	49, 98%

**Table 2.** ICU Population: Intubated vs Non-Intubated

	**Overall**	**ICU Non-Intubated**	**ICU Intubated**
Encounters	50	30	20
Patients	48	28	20

**Demographics**			
Mean Age (years)	61.9	63.0	60.2
Sex	M, 24, 50%	M, 12	M, 12
	F, 24	F, 16	F, 8
Race			
Black	32	15	17
White	14	11	3
Other	4	4	0

**Etiology of AE**			
	ACEi, 27	ACEi, 17	ACEi, 10
	Unknown, 15	Unknown, 9	Unknown, 6
	Other, 6	Other, 3	Other, 3
	Hereditary, 2	Hereditary, 1	Hereditary, 1

**Mean LOS (days)**			
Total Hospital LOS	3.37	1.55	6.15
ICU LOS	2.40	1.10	4.35

**Comorbidities**			
Airway (OSA, COPD, Asthma)	12	6	6
Prior AE episodes or diagnosis of Hereditary AE	11	8	3

## Results

We identified 135 encounters for angioedema among 117 total patients (Table 1). Of note, Patients were evenly split based on sex (Male: 63, 55%) and had a mean age of 58.5 years old. Most patients identified as Black/African American (80, 68%). The top etiologies for angioedema recorded were the following; Unknown (64, 47.4%), ACEi (51, 37.8%), and Hereditary (6, 4.4%). For treatment, 78.4% received a histamine, corticosteroid, epinephrine regimen, 8.5% received no pharmacotherapy, and 1% received a biologic agent (Icanibant). The average total hospital LOS was 1.43 days with 93% of encounters discharged home.

Of the 135 encounters, 50 were admitted to an ICU setting (either medical, surgical, or cardiovascular) while the other 85 were managed outside of the ICU setting. 48 of the 50 ICU encounters were admitted directly to an ICU, while 1 was admitted from the ED observation unit and one from a medicine service respectively.

**Fig. 2.**
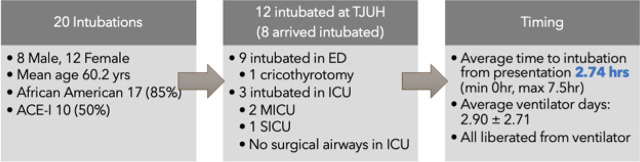
Figure describing patient demographics of the 20 intubations in our study, intubation location, and intubation timing and other data collected.

Twenty of the 50 encounters were intubated (ICU-intubated group) while the other 30 did not require intubation (ICU-nonintubated group) (Table 2 and 3). Intubations mainly occurred outside of the ICU (9 of 12 at TJUH) or before transfer to our institution (8). Only 3 intubations occurred in an ICU setting: 2 in the medical ICU and 1 in the surgical ICU. One patient required a cricothyrotomy, which was performed in the ED by our ENT team. The mean time from presentation to intubation was 2.74 hours (Min 0hr; Max 7.5hr). There was an average of 2.9 ventilator days after intubation and all patients were successfully liberated from the ventilator (Figure 2). No deaths occurred. One patient in this study possessed a chronic tracheostomy and did not require ICU stay, they were excluded from the intubation group.

**Table 3.** Characteristics of Intubated Patients and Intubation Information

**Intubated Patients**	**Sex**	**Age**	**Race**	**Angioedema Etiology**	**Intubation Location**	**Intubation Timing from Presentation**	**Intubation rationale**	**Comorbidities/PMH**
**1**	F	57	AA	ACE-I	ED	2.75	Dysarthria, tongue edema, floor of mouth edema	HTN, asthma, COPD, breast cancer
**2**	F	77	AA	ACE-I	ED	1.5	Diffuse edema	T2DM, HTN Obesity
**3**	M	51	AA	ACE-I	ED	2	Tongue edema, floor of mouth edema	T2DM
**4**	M	63	AA	ACE-I	ICU	7	Thickness of epiglottis, tongue edema, buccal mucosa edema	Prostate cancer, HTN
**5**	M	37	AA	ACE-I	ED	1.3	Tongue edema, floor of mouth edema, thickness of epiglottis	Obesity, CVA, HTN
**6**	M	68	AA	ACE-I	ED	2	Thickness of epiglottis	HF, AVr, COPD
**7**	F	61	W	HAE	ED	3.25	Diffuse edema, thickness of epiglottis, buccal mucosa edema	Hereditary Angioedema
**8**	F	30	AA	ACE-I	ICU	7.5	dysphagia, dysphonia, uvular swelling	HTN
**9**	F	74	AA	Methotrexate	ED	0	Dysphagia, diffuse edema, dysarthria	SLE, RA, GERD, HTN, DM, HLD, CAD, CKD, OSA, HFpEF
**10**	M	48	AA	Unk	OSH	66*	Buccal mucosa edema	HF, CKD III, T2DM, COPD
**11**	M	40	AA	Unk	ED	3	Diffuse edema, intolerance of secretions, thickness of epiglottis	Angioedema, CVA, HTN, T2DM, HLD
**12**	F	72	AA	Unk	ICU	1.5	Diffuse edema	HTN, OSA, HAE, prior trach 2016
**13**	M	69	W	Unk	ED	1.25	Diffuse edema, intolerance of secretions	ESRD, SUD, Angioedema
**14**	M	67	AA	Unk	OSH	2.5	Diffuse edema, dysarthria	HIV, COPD, Anal Cancer, T2DM, CKD, CAD
**15**	M	68	W	ACE-I	OSH	-	-	CABG, DM, HLD, Bladder cancer, HTN
**16**	F	55	AA	ACE-I	OSH	-	-	HTN, G6PD, HLD
**17**	M	57	AA	ACE-I	OSH	-	-	HTN, Seizures, Gout
**18**	M	65	AA	ACE-I	OSH	-	-	Renal Transplant, HTN, SUD
**19**	F	84	AA	Allergy	OSH	-	-	Dementia, HTN
**20**	M	61	AA	Unk	OSH	-	-	Angioedema, CVA, HTN, T2DM, HLD

As for significant differences between groups, we explored various factors such as comorbidities, ICU and hospital LOS, etiology, and demographics. There were 6 patients with airway comorbidities in the ICU-intubated group (defined as a history of obstructive sleep apnea, chronic obstructive pulmonary disease, or asthma) which was the same as the ICU-nonintubated group. There were 3 patients who carried a diagnosis of hereditary angioedema or had prior angioedema episodes documented in the ICU-intubated group, which was fewer than the 8 patients identified in the ICU-nonintubated group. Mean ICU LOS for the ICU-nonintubated group was 1.1 ± 0.6 days, with a mean total hospital LOS of 1.6 ± 1.4 days compared to 0.3 ± 0.7 days for angioedema patients not admitted to an ICU setting (such as ED Observation or medical surgical floor) (p<0.001)(CI;-8.5, 15.2). Mean ICU LOS for intubated patients 4.3 ± 3.2 days with a mean total hospital LOS of 6.2 ± 4.9 days (Figure 3). Those with ACEi angioedema were more likely to be admitted to the ICU (OR 2.46, CI 95%, (1.19–5.05)) and those with unknown etiology were less likely to be admitted to the ICU (OR 0.33 CI 95% (0.16–0.69)). While there were no significant differences in etiology between the ICU-intubated and ICU-nonintubated groups, African Americans were significantly more likely to be intubated as compared to other races (OR 5.67 95% CI (1.37 – 23.46)).

**Figu. 3.**
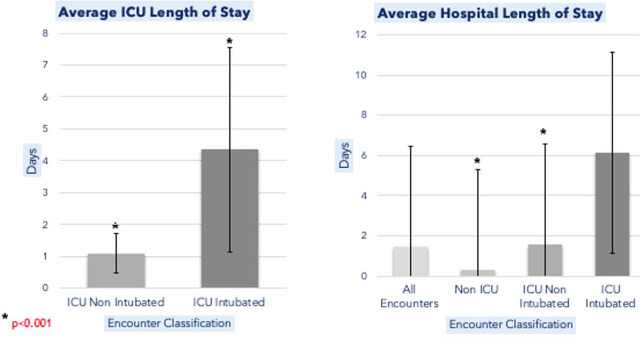
(Right) Average ICU Length of Stay in days compared between the ICU Non Intubated and ICU Intubated patient groups with * representing a p-value of <0.001. (Left) Average Hospital Length of Stay in days compared between all subset groups.

## Discussion

With these results, we confirmed that at our institution, angioedema patients are often intubated early in their hospital course and outside of the ICU setting. At TJUH, 14% of patients presenting with angioedema in our study were intubated in the ED. This was an equivalent intubation rate compared to available literature for intubation rate of angioedema patients in the ED, suggesting that our initial management is neither more aggressive or conservative than typical management. For patients not requiring immediate intervention, it becomes a less clear picture for who requires observation at a higher level of care like an ICU. Our institution lacks a defined set of criteria for which patients should be sent to the ICU for “airway watch” and is often decided on a case-by-case basis that has demonstrated inconsistent patterns of ICU admission for angioedema patients such as stable patients with no concerning exam or NPL findings. It is cautious to keep patients in a controlled environment like the ICU in the event of airway collapse or need for an emergent airway, however it is not without consequence. We demonstrated that several patients that had an unnecessary extension of their ICU and hospital stay for “airway watch” as compared to their non-ICU counterparts. With these findings, we propose that patients not intubated in the first few hours of their hospital stay may be monitored outside of the ICU to shorten their LOS and conserve ICU resources [1,2,8].

When assessing possible clinical decision aides to make these judgements, we considered common characteristics or patterns in our cohort among intubated angioedema patients. There were few differences between the ICU-intubated and ICU-nonintubated group. However, it was noted that ACEi angioedema encounters were more likely to be admitted to the ICU. ACEi angioedema has similarly lead to higher ICU admission rates in other studies. The incidence of ACEi angioedema has increased as ACEi prescriptions have become more common. ACEi angioedema is a subset of bradykinin-mediated angioedema, it often affects the lips, tongue, oral cavity, and larynx which can make intubation very difficult. It also has fewer options for treatment, such as corticosteroids and antihistamines, as compared to other etiologies of angioedema. With this information, it is understandable that these patients were treated with more caution. However, we observed no difference in etiology between our intubated and non-intubated group. While these patients may have strong reason for admission due to risk of airway collapse and difficult intubation, they may not need to be observed in the ICU setting if they are stable and may be more suitable for an ED observation unit or step-down unit [1,4,6, 9].

It was also observed that in the ICU, African Americans were more likely to be intubated as compared to other races. There are many studies that have identified higher incidence rates of angioedema among African Americans. Brown et al. found that although black and white patients with ACEi were equally admitted to the hospital, there were a higher proportion of black subjects that required ICU care and intubation suggesting that not only is incidence higher, but severity is worse in this population. There is no known mechanism for these findings, but it has been proposed that there are race-related differences in the kallikrein-kinin system that may play a role. There is more research needed to determine why we observe higher morbidity and mortality in the African American population [4, 9,10,11].

Limitations of our study include our small population size limited by and setting of a single urban quaternary referral center with the availability multiple ICU settings and access to ENT specialists. ENT at our institution is responsible for serial monitoring of patients with nasopharyngeal laryngoscopy (NPL) both outside and inside of the ICU until the patient improves or requires intubation. We also have a Compromised Airway Response Team (CART) that includes members of Anesthesia, ENT, and Trauma Surgery that report to emergent airways throughout the hospital. Additionally, this study was retrospective and nonblinded, which may result in selection bias.

It is also important to discuss that while our findings suggest that stable angioedema patients may be safely monitored outside of the ICU, there are potential risks of not admitting these patients to a higher level of care. The decision to forego ICU admission must carefully balance resource allocation with patient safety. Failure to identify patients at risk for airway collapse may lead to delayed interventions, increase morbidity, and potentially fatal outcomes. Future studies should be aimed at defining appropriate risk stratification tools and exam criteria, such as NPL findings as demonstrated in Ischoo et. al and Gayen et. al. or the use of serial NPL exams that are performed at our institution, to ensure that patients who genuinely require ICU-level monitoring receive appropriate care while minimizing unnecessary ICU admissions.

## Conclusion

In a small cohort of angioedema patients presenting to a quaternary referral center with ENT available, airway decompensation and intubation occurred early in the hospital course and mostly outside of the ICU. We suggest that angioedema patients who have remained stable after the first few hours of their hospital stay in similar settings and are being considered for “airway watch” are safe to be monitored outside of the ICU setting. Benefits include shortening the overall LOS for these patients and conservation of ICU resources. Further research is needed to elucidate which AE patients and etiologies are more likely to result in higher morbidity and mortality. We hope this study will help guide creation of a formal criteria for angioedema “airway watch” in the ICU at our institution attempting to limit unnecessary use of ICU resources.
